# Community health worker outreach to farmworkers in rural North Carolina: Learning from adaptations to the SARS‐CoV‐2 pandemic

**DOI:** 10.1111/hex.14047

**Published:** 2024-04-13

**Authors:** Catherine E. LePrevost, Leslie E. Cofie, Julianna Nieuwsma, Emery L. Harwell, Natalie D. Rivera, Paula A. Acevedo, Joseph G. L. Lee

**Affiliations:** ^1^ Department of Applied Ecology, College of Agriculture and Life Sciences NC State University Raleigh North Carolina USA; ^2^ Department of Health Education and Promotion, College of Health and Human Performance East Carolina University Greenville North Carolina USA; ^3^ NC Farmworker Health Program, Office of Rural Health NC Department of Health and Human Services Raleigh North Carolina USA

**Keywords:** COVID‐19, community health workers, community‐institutional relations, farmers, transients and migrants

## Abstract

**Background:**

Community health workers represent a critical part of the health outreach and services for migrant and seasonal farmworkers (‘farmworkers’) in rural areas of the United States.

**Purpose:**

We sought to identify adaptations to farmworker patient engagement and health outreach made by community health workers during the first 18 months of the COVID‐19 pandemic.

**Methods:**

In this qualitative study, we used semi‐structured interviews with community health workers from August 2020 to February 2022 (*n* = 21). Two coders used thematic analysis to identify three themes related to the experiences of community health workers in conducting health education and outreach to farmworkers prior to and following the onset of the pandemic.

**Findings:**

We found themes related to pre‐pandemic outreach efforts to provide health education resource sharing with farmworkers and pandemic‐related outreach efforts that included adoption of porch drops and distanced delivery of health education, adaptation of modes of health education and communication through technology and the internet, and taking on new roles related to COVID‐19. Finally, we identified changes that reverted after the pandemic or will continue as adaptations.

**Conclusions:**

Community health workers created practice‐based innovations in outreach in response to the COVID‐19 pandemic. These innovations included new COVID‐19 related roles and new modes of health education and outreach, including the use of digital resources. The changes developed for emergency use in COVID‐19, particularly related to internet and technology, have likely altered how community health workers conduct outreach in North Carolina going forward. Funders, community health worker training programs, and researchers should take note of these innovations.

**Patient or Public Contribution:**

Community health workers who typically come from patient populations and provide critical navigation and connection with the health care system advised on the design and creation of this research project, including serving on an advisory board. Two authors have experience working as community health workers.

## 1 INTRODUCTION

Community health workers are a key part of the rural health infrastructure, particularly for reaching agricultural workers such as migrant and seasonal farmworkers (‘farmworkers’).^1^ Community health workers originated as a health promotion model in rural 1920s China,^2^ which is used worldwide to promote health in rural areas^2^ and represents an important strategy to address health equity.^1^ Farmworkers in the United States work predominantly in rural areas and face substantial health inequities and structural vulnerabilities, including barriers to accessing health care based on language and geography.^3^ COVID‐19 exacerbated these challenges, and death rates from COVID‐19 have been documented as higher among more vulnerable workers.^4^, ^5^ A critical piece of the community health worker model is having a shared language, culture, and background that can facilitate building trust and understanding through personal connections.^4^, ^5^

The COVID‐19 pandemic provided a unique challenge to this model as it required rapid changes to community health workers’ outreach to farmworkers, including pausing in‐person outreach and stymying personal connections.^6^, ^7^ Prior reviews have shown the importance of community organizations and outreach in pandemic response.^8^ Additionally, prior research from the field of social work has explored changes in outreach to farmworkers in rural Michigan, finding pauses in in‐person outreach, increased use of social media, provision of wi‐fi, and use of personal protective equipment (PPE).^9^ Other researchers have documented state and interagency coordination efforts^6^ and emergency provision of internet access as part of COVID‐19 response for farmworkers.^10^ Yet, little research has worked with community health workers to bring their voices and descriptions of practice‐based^11^ innovations from COVID‐19 response into the literature.

The Community Health Worker Core Consensus Project has identified core community health worker roles, including providing culturally appropriate health education and information and conducting outreach, and core skills, including interpersonal and relationship‐building skills and service coordination and navigation skills.^12^ While community health workers are not licensed, community health worker associations and certification programs are under development in the United States.^13^ In North Carolina, farmworker‐serving community health workers are employed by federally funded migrant and community health centers, local health departments, free and charitable clinics, and non‐profit organizations. They perform a critical role in farmworker healthcare by developing relationships with farmworkers, conducting health assessments, providing health education and case management, and connecting farmworkers to medical providers.^5^ Prior research with farmworker‐serving community health workers in North Carolina has shown that most identify as being Latina women and have completed high school or college as their highest level of educational attainment.^14^

Given the potential for future pandemics and the ongoing challenges of COVID‐19, it is critical to understand how community health workers transformed health education and outreach models in response to the pandemic and to document practice‐based innovations in response to these changes. Thus, we sought to capture how community health workers adapted to the changing conditions brought on by the pandemic and how these adaptations continue in their work.

## 2 METHODS

We conducted a thematic analysis of interviews with 21 community health workers. The interviews came from the process evaluation of three projects related to digital inclusion for agricultural workers in North Carolina during the onset and/or early stages of the pandemic. Each project sought to address the digital divide faced by farmworkers and their families by providing technological resources and related skills for internet access and use. Participants were recruited with assistance from our community partners through referrals of project participants who were receiving assistance or training.

We conducted interviews by phone or videoconference between August 2020 and February 2022 in English or Spanish. Community health workers represented education (e.g., Migrant Education Program) and health center employees. Interviews lasted approximately 30 min. More information on individual projects can be found in preliminary reports and news coverage.^10^, ^15^ Interview guides, which are available in our institutional repository,^16^ focused on services provided to farmworkers, implementation of internet access interventions, and future challenges and sustainability. COVID‐19 was not explicitly a topic in the interview guide.

Despite the lack of focus on COVID‐19, the 21 community health workers (see Table 1) found it difficult to disentangle COVID‐19 from their accounts of participation in the various projects. Some asked if they were supposed to talk about ‘before and after [the pandemic]’, and many went ahead and did just that. COVID‐19 arose naturally in conversation with the interviewees, which prompted the development of deductive codes to track how COVID‐19 disrupted regular activities (i.e., how outreach was conducted before COVID‐19) and how outreach changed over the first 18 months of the pandemic.

**Table 1:** Participant demographics, North Carolina, *n* = 21, August 2020 to February 2022.

Demographic characteristic	Response category	*n* (%)
Gender	Male	3 (14)
	Female	14 (67)
	Not reported	4 (19)
Latine/Hispanic or Non‐Hispanic	Latine/Hispanic	16 (76)
	Non‐Hispanic	1 (5)
	Not reported	4 (19)
Self‐reported identity	Black or African American	‐
	Hispanic/Latino	6 (29)
	White	3 (14)
	Other response	6 (29)
	Not reported	6 (29)
Education level	<High school	‐
	High school	1 (5)
	Some college	1 (5)
	Associate's	3 (14)
	Bachelor's	9 (43)
	Master's	2 (10)
	Not reported	5 (24)

Thematic data analysis began with initial close readings of the transcripts and used NVivo 12/PC. Four researchers worked to develop a preliminary codebook, which included the deductive codes derived from the interview guide and inductive codes obtained from line‐by‐line coding of four transcripts.^17^ The researchers met regularly to refine the codebook and ensure reliability. Our code output summaries revealed codes related to COVID‐19 and COVID‐related adaptations or reflections. Thus, we used a ground theory approach to examine distinct COVID‐related themes based on the experiences of community health workers. This framework enabled us to assess community health workers’ health outreach prior and during the pandemic, with a particular focus on how COVID impacted their outreach work. The research procedures were reviewed by the East Carolina University and Medical Center Institutional Review Board and approved with an exempt determination (#19‐001817).

## 3 RESULTS

Findings from interviews with community health workers indicated that most participants reported being female (67%) and Latine/Hispanic (76%) and having an associate's degree or higher (67%). Community health workers’ descriptions of their involvement in health education and outreach before, during, and after the pandemic revealed three salient themes: pre‐pandemic health education and outreach efforts; pandemic‐related health education and outreach efforts, including health education and outreach efforts tackling COVID‐19; and expansion of future health education and outreach. Below, we describe each theme and include quotations to highlight the experiences and perspectives of community health workers.

### 3.1 Pre‐pandemic health education and outreach

Before the COVID‐19 pandemic, community health workers described hosting gatherings at housing sites to provide health education and share resources with farmworkers. These gatherings, typically ranging in size from 3 to 30 workers, often occurred during the evening after workers completed their workday. During these gatherings, community health workers described sharing videos on projector screens, leading group discussions or activities, and passing out educational pamphlets on various health topics and resources. Several participants described how their organizations followed a three‐visit model: first carrying out basic health assessments and patient registration, next doing point‐of‐care labs, and then responding to specific needs observed during the first two visits, including bringing a health care provider or taking the patient to the clinic. Community health workers provided additional services to farmworkers, including the provision of hygiene and first aid kits, transportation to health clinics and pharmacies, picking up prescriptions or other needed items from stores and delivering them, signing eligible farmworkers up for health insurance, and providing referrals to legal services or the department of labor when needed.

Community health workers’ use of technology as a part of their jobs was limited mainly to computer usage at the office and occasionally communicating with farmworkers through cell phones (e.g., text messages and WhatsApp). Health education primarily took place in person and relied heavily upon printed materials. Educational content often focused on preventive health topics and illnesses common within the farmworker community (e.g., heat stroke, diabetes). One participant explained,

Our health education modules are just a folder with some pamphlets and brochures, and we touch on heat illness, alcohol, mental health, nutrition, and other topics related to farmworker health. It's, of course, presenting what's on paper but also getting that conversation started, engaging them because just reading out loud what's on a sheet of paper is not really helpful. (Participant 2)

### 3.2 Adaptations to health education and outreach during the pandemic

#### 3.2.1 Subtheme: Adoption of porch drops and distanced delivery of health education

In the early days of the pandemic, community health workers were unable to gain physical access to the farmworkers' housing units because of clinic or organization polices to limit COVID‐19 transmission. Standard practices of a three‐visit model were halted due to safety risks for community health workers, farmworkers, and their families. One participant shared, ‘During this past year, because everything was about COVID, there were many things that we weren't able to do because of the social distancing and not risking or exposing ourselves to the virus’ (Participant 15). When community health workers were ready to resume in‐person visits, they encountered challenges accessing housing locations when employers limited visits to mitigate exposure risk. As described by Participant 24, ‘We don't have access to the camps because of COVID. The growers have reserved those rights. We had to get like Legal Aid and different people involved just so that the growers know that we're there to provide assistance and aid to them and to the workers’. Community health workers instead engaged in ‘porch drops’ to deliver food, personal hygiene supplies, medication, and PPE kits. They incorporated health education into these ‘porch drops’ by leaving pamphlets containing COVID‐related information. One participant described,

We did take that time to help the farmworkers stay up to date on all the COVID information that the CDC was putting out. We printed information, and we would laminate, and we would still visit them and just leave them that information and then also we received some PPE from the state, and so we were able to make little bags for them with PPE disposal face masks, cloth face masks, some hand sanitizer and some gloves and so we distributed that to the farmworkers as well. (Participant 15)

In light of these constraints, participants expressed that their outreach efforts felt rushed, as they were unable to spend as much time with farmworkers.

#### 3.2.2 Subtheme: Adapting modes of health education and communication through technology and internet

Community health workers also increasingly leveraged technology to continue providing outreach services to farmworkers during the pandemic. Participants explained that their means of health education outreach became more oriented toward digital sources. They provided virtual education opportunities through a variety of internet‐based platforms (e.g., Zoom, WhatsApp, Facetime). This included live‐streaming events, phone calls, and sharing documents and files containing educational materials. One participant stated: ‘As DHHS or the CDC come out with more Spanish material, we're uploading that on our WhatsApp story or our Facebook page’ (Participant 2). Also, telehealth appointments for both health education and medical appointments became more prominent during the pandemic, and, for some, were allowed for the first time:

Before COVID, we weren't allowed to do any health assessments or assess any needs on the phone. It always had to be a face‐to‐face encounter. Now, we're able to do health assessments through phone, through WhatsApp, or through FaceTime, or a video messenger. (Participant 5)

Though community health workers communicated with farmworkers via cellular phone before the pandemic, reduced in‐person contact led to a greater reliance on digital modes of contact, health education, and service provision during the pandemic.

The use of digital resources and telehealth services was possible for many farmworkers and their families because of expanded access to the internet. During the initial pandemic shutdown, community health workers were able to communicate only with farmworkers who had cellular phone data. Internet connectivity, via hotspots in many farmworker housing units, helped overcome challenges with contacting farmworkers and ensured their access to healthcare. In instances when farmworkers felt, as Participant 2 indicated, ‘“Oh, well, my data plan ended’, or, ‘I can't go to the store, so I can't add more minutes to my phone’”, they were still reachable by community health workers. One community health worker mentioned, ‘And so we gave them the hotspots and that's how we were able to continue checking their vitals and making sure that they were doing well. And they're still using [hotspots] right now, and it's a huge, huge benefit’ (Participant 2).

Access to the internet in conjunction with technology resources enhanced the quality and timeliness of services offered to farmworkers by community health workers. For instance, community health workers did not ‘have to wait to come back to the office to get certain [health] information’ to address questions posed by farmworkers, including questions clarifying information farmworkers themselves found online (Participant 15). Technology resources, such as large projector screens, megaphones, and tablets to document health forms and download videos, enabled health education and outreach while facilitating distancing between community health workers and farmworkers and among groups of farmworkers. As one community health worker succinctly noted, ‘Everyone was able to spread out’ (Participant 24).

#### 3.2.3 Subtheme: New roles related to COVID‐19

Once the pandemic began, community health workers turned their attention away from their standard preventative health curricula (e.g., diabetes management and prevention, heat illness), to focus on curtailing the rapid spread of COVID‐19. They were tasked with guiding farmworkers through testing, contact tracing, isolation, and vaccinations. One participant described the shift in focus to COVID‐19: ‘Before the pandemic, I was checking blood pressure, asking them about their health, more general health problems, but now I am more focused on everything related to the pandemic’ (Participant 6). Some participants described being asked by other agencies to do testing/vaccine clinics outside of their typical service areas. Staying educated and current on COVID‐19 was stressful for community health workers due to frequent changes in official recommendations for prevention and management.

### 3.3 Health education and outreach work going forward

Thinking beyond the initial phases of the pandemic, almost all community health workers expressed their intent to resume in‐person visits to farmworkers, particularly to facilitate interactive presentations with demonstrations and Q&As. Several community health workers described their plans to reintroduce general health topics (e.g., preventive health and work‐related illnesses) during outreach and health education. One participant explained, ‘…[H]eat illness, nutrition, and mental health—we haven't touched much on that…as we hope for and how we usually did just because of COVID, and we've been prioritizing COVID health education and so hopefully this year we are able to jump back into demonstrating some health education with the other topics that are not COVID related’ (Participant 2).

A subset of community health workers described their intentions to continue using technology and the internet into the future. Community health workers emphasized the importance of continuing hotspot lending programs. For example, one community health worker stated, ‘The farmworkers themselves were so grateful, and they were talking about the next year “Oh, maybe if we all pitch in five dollars, we can get internet if we don't get these hotspots again” and so they saw the benefits in that, and they were able to access a lot of health education’ (Participant 2). The same community health worker shared plans for using digital resources in new ways, ‘We really wanted to do group WhatsApp calls where we call some of them and then three or four guys, we can give them a health education session. That hasn't happened yet, just given that we're handling a lot of other things. But that's one thing that we are interested in as well’ (Participant 2). Another community health worker's organization found using tablets distributed during the pandemic to be so effective that the finance department purchased more so that they could ‘transition completely to doing everything electronically’ (Participant 15). Community health workers predicted that incorporating digital resources, such as hotspots and social media, into their work would assist with providing more reliable follow‐ups, which would in turn aid in building stronger trust with farmworkers.

## 4 DISCUSSION

This study revealed the adaptations community health workers developed and implemented during the first 18 months of the COVID‐19 pandemic while conducting health education and outreach to farmworkers in rural North Carolina. Much of the participating community health workers' pandemic response relied upon interagency coordination, which has been emphasized in COVID‐19 response in urban environments.^18^ We found that community health workers adopted new modes for continuing their principal functions of health education and outreach, including the use of digital resources. Additionally, community health workers took on new roles specifically related to COVID‐19, including the support of testing, contact tracing, and later vaccinations for farmworkers.

Community health workers provided critical aid to farmworkers during the pandemic. PPE provided by the community health workers in our study was likely important for limiting virus spread within and between housing sites. PPE provision might also have served to mitigate some of the well‐documented chronic health illnesses and structural vulnerabilities experienced among farmworker communities, which can be exacerbated by the virus spread.^19^, ^20^ Other studies have also reported that community health workers played critical roles in distributing PPE supplied from government aid packages to farmworkers.^9^, ^21^ In addition to a lack of access to PPE, large portions of the Hispanic population in the United States faced food insecurity during the pandemic^22^ due to isolation, transportation issues, and economic hardship. Porch drops from the community health workers who participated in our study provided needed food and prescriptions to farmworkers who were isolated in rural housing sites.

While prior research indicates that farmworkers are receptive to virtual healthcare,^23^ our participants indicated that virtual health education and outreach were not common before COVID‐19 and were sometimes even prohibited by their employing organizations. Our study suggests this changed in North Carolina as community health workers and their organizations mitigated risks associated with in‐person visits, reflecting telehealth trends throughout the United States during the pandemic.^24^ Internet‐based platforms, such as WhatsApp and Facebook, emerged as one of the primary forms of maintaining communication and disseminating health information to farmworkers during the pandemic. This finding aligns with limited research on outreach to farmworker communities in other states during this time period.^9^, ^21^ Shaver, however, emphasizes that telehealth should be a supplement for health care, not a replacement,^24^ and community health workers' plans for future health education and outreach support this belief. The participating community health workers emphasized the importance of reinstating interactive and participatory health education; community health workers have previously described this format as preferrable to other available formats.^14^ Participants also expressed a desire to augment their in‐person activities with technology and digital resources. Future research should consider the optimal balance of in‐person and virtual activities in providing services to farmworkers.

A strength of the study is that it provided information about the experiences of community health workers in their health outreach efforts during the pandemic. However, there are a few limitations. First, we did not set out to research responses to COVID‐19, and so our interview guide was not specifically designed to assess adaptations. Second, we conducted interviews during COVID‐19, and this may have influenced how participants remembered outreach before the pandemic. Third, this project took place in the context of a project on digital inclusion, and this may have influenced how participants adapted to COVID‐19. Additionally, the findings may not be generalizable to community health workers across the United States, as our participants were community health workers in North Carolina.

## 5 CONCLUSIONS

Community health workers are a critical part of farmworker health services in rural, agricultural areas. This paper shows the resilience, adaptability, and value of community health workers in addressing farmworker health in the context of a pandemic. Community health workers were able to provide farmworker communities with the goods, information, and services they needed through porch drops, live‐stream events, social apps, and partnering with other agencies. Additionally, this paper shows how practitioner adaptations of outreach models helped contribute to COVID‐19 response. COVID‐19 changed how community health workers in North Carolina utilize technology, and innovations from that change continue even as the COVID‐19 pandemic has changed to an endemic problem. Researchers and funders should consider the importance of technological literacy, in a post‐pandemic world, as an area for continuing research and development and assess community health worker outreach models for innovations created by the participants in this study. Ensuring support for and engagement with community health workers can help advance health services in rural areas.

## AUTHOR CONTRIBUTIONS

**Catherine E. LePrevost**: Conceptualization; funding acquisition; writing—original draft; investigation; methodology; writing—review and editing; supervision; resources. **Leslie E. Cofie**: Conceptualization; writing—original draft; writing—review and editing; methodology; formal analysis; supervision. **Julianna Nieuwsma**: Formal analysis; writing—review and editing; writing—original draft. **Emery L. Harwell**: Writing—original draft; writing—review and editing; formal analysis. **Natalie D. Rivera**: Writing—review and editing; resources. **Paula A. Acevedo**: Investigation; writing—review and editing. **Joseph G. L. Lee**: Writing—review and editing; funding acquisition.

## CONFLICT OF INTEREST STATEMENT

The authors declare no conflict of interest.

## Data Availability

The data that support the findings of this study are available on request from the corresponding author. The data are not publicly available due to privacy or ethical restrictions.
